# Hybrid Neuro-Symbolic State-Space Modeling for Industrial Robot Calibration via Adaptive Wavelet Networks and PSO

**DOI:** 10.3390/biomimetics11030171

**Published:** 2026-03-02

**Authors:** He Mao, Zhouyi Lai, Zhibin Li

**Affiliations:** 1 School of Sino-German Robotics, Shenzhen University of Information Technology, Shenzhen 518172, China; 2 School of Software Engineering, Chengdu University of Information Technology, Chengdu 610225, China

**Keywords:** industrial robot calibration, neuro-symbolic state-space model, meta-optimization, adaptive wavelet network, kinematic parameter identification

## Abstract

The absolute positioning accuracy of industrial manipulators is frequently bottlenecked by the interplay of geometric tolerances and complex, unmodeled non-geometric parameter drifts. Traditional static kinematic models, predicated on rigid-body assumptions, often struggle to characterize these state-dependent dynamic behaviors. To bridge this gap, this study introduces a PSO-Driven Neuro-Symbolic State-Space Framework incorporating Adaptive Wavelet Networks, drawing inspiration from two biological principles: the collective swarm intelligence observed in bird flocking and fish schooling, and the localized receptive field structure of mammalian visual cortex neurons. By reformulating calibration as a latent state estimation problem, we model kinematic parameters as stochastic states. Crucially, the observation model fuses symbolic Denavit–Hartenberg (D–H) predictions with an Adaptive Wavelet Network (AWNN). The AWNN utilizes Mexican Hat kernels, whose morphology mirrors the center-surround antagonism of cortical receptive fields, and leverages their precise time–frequency localization to effectively learn complex, configuration-dependent residuals. The framework employs a robust decoupled strategy. First, Particle Swarm Optimization (PSO) executes meta-optimization to autonomously determine hyperparameters, thereby mitigating initialization sensitivity. Second, a recursive inference engine estimates the hybrid states. Third, a global batch optimization refines the symbolic parameters against a frozen non-geometric error field. Experimental validation on an ABB IRB 120 robot (400 datasets) yielded a test RMSE of 0.73 mm. Compared to the standard Levenberg–Marquardt method, our approach reduced the RMSE by 40.16% and the maximum error by 35.71% (down to 0.99 mm). Moreover, it outperforms the state-of-the-art RPSO-DCFNN baseline by 12.05% while maintaining high computational efficiency (convergence within 20.15 s). These findings underscore the superiority of the proposed bio-inspired state-space fusion strategy for high-precision industrial applications.

## 1. Introduction

With the rapid advancement of industrial automation, industrial robots have become ubiquitous in the era of intelligent manufacturing [1]. By virtue of their high safety, strong versatility, and exceptional efficiency, industrial robots have become core equipment in modern production [2]. They are widely used for highly repetitive, hazardous, and technically complex tasks in logistics, aerospace, and medical care. However, despite their distinct advantages, absolute positioning accuracy [3] remains a critical bottleneck. Manufacturing tolerances [4], assembly errors, and structural compliance often cause deviations between the theoretical and actual pose. This issue becomes more pronounced in high-precision machining, where motion control is required at extremely fine scales [5]. As the errors accumulate, robots may fail to reach predetermined positions accurately, which compromises process stability and product quality.

To mitigate these issues, robot calibration technology [6] has emerged as a primary solution. Calibration improves accuracy by identifying systematic geometric and kinematic parameter errors and compensating for them. Currently, high-precision measurement instruments such as laser trackers [7], ball bars [8], and electronic theodolites [9] are widely used. These devices offer high accuracy, large measurement ranges, and strong anti-interference capability. However, they are prohibitively expensive and typically require professional training. As a result, they are often impractical for small and medium-sized enterprises (SMEs). Consequently, achieving a balance between calibration effectiveness, cost reduction, and ease of use has become a key research objective.

In this context, draw-wire encoders [10] present a cost-effective alternative to expensive measurement instruments due to their simple structure and flexibility. However, they can be sensitive to environmental disturbances, which limits their direct use in complex industrial scenarios. This motivates calibration algorithms that are both robust and computationally efficient for low-cost sensing. In recent years, data-driven approaches, particularly neural networks, have been widely applied to address these challenges. Xu et al. [11] used a back-propagation neural network (BPNN) to optimize controller angle inputs and compensate for joint flexibility and geometric errors. Gao et al. [12] further integrated BPNN with particle swarm optimization (PSO) to improve global search and identify geometric parameters. In medical robotics, Hwang et al. [13] employed a recurrent neural network (RNN) to model hysteresis and nonlinear errors in the da Vinci surgical robot. Chen et al. [14] proposed an error-compensation method that combines a radial basis function neural network (RBFNN) with error similarity analysis. Related studies also applied neural models to account for non-geometric factors [15,16], and Le et al. [17] developed a network structure to constrain maximum positioning deviations.

Despite the significant accuracy improvements reported in these studies, neural-network-based schemes face inherent limitations. They often behave as black-box models with limited physical interpretability, require large datasets for training, and may overfit when modeling the nonlinear, configuration-dependent dynamics of robot manipulators. Traditional model-based approaches also face challenges. Pure extended Kalman filter (EKF) methods enable online Bayesian filtering but can suffer from linearization errors. In contrast, the Levenberg–Marquardt (LM) algorithm is effective for batch optimization but lacks an explicit mechanism to weight measurement noise.

To overcome these limitations, this paper proposes a PSO-driven neuro-symbolic state-space framework with adaptive wavelet networks, drawing on two bio-inspired principles. First, the collective swarm intelligence observed in bird flocking and fish schooling motivates the use of Particle Swarm Optimization (PSO) for robust hyperparameter meta-optimization. Second, the center-surround antagonism of mammalian cortical receptive fields inspires the adoption of Mexican Hat wavelet kernels, which replicate this localized excitatory-inhibitory structure to selectively capture configuration-dependent residual singularities. The proposed method targets two key issues: (i) static models struggle with complex non-geometric errors, and (ii) conventional black-box neural networks lack local resolution. This strategy bridges the gap between physics-based kinematics and data-driven dynamics learning. Specifically, we reformulate the calibration problem as a latent state estimation process within a hybrid state-space model. The symbolic D–H kinematics module ensures physical interpretability, while the Adaptive Wavelet Network (AWNN) module leverages time–frequency localization to explicitly learn configuration-dependent residuals in the observation space. The proposed method follows a decoupled three-stage strategy. First, a PSO-driven meta-optimization layer searches for suitable initial hyperparameters to reduce sensitivity in recursive estimation. Second, a recursive EKF engine jointly estimates stochastic geometric states and wavelet residuals. Third, based on these priors, a global Levenberg–Marquardt batch optimization [18] refines the symbolic parameters using a frozen non-geometric error field. This hybrid approach achieves a superior balance between dynamic error compensation and global geometric precision. The main contributions of this paper are summarized as follows:

- **Neuro-symbolic state-space calibration with adaptive wavelet residual learning:** We develop a calibration framework that fuses symbolic D–H kinematics with an AWNN residual model in a unified state-space formulation, capturing configuration-dependent non-geometric effects while preserving physical interpretability.
- **PSO-driven meta-optimization for robust initialization:** We introduce a PSO layer to automatically select key hyperparameters and initialization settings, reducing the risk of filter divergence and improving robustness.
- **Decoupled refinement for stable global convergence:** We decouple residual learning from geometric refinement by freezing the learned non-geometric field and then refining symbolic parameters via LM, which accelerates convergence and improves calibration accuracy (RMSE reduced to 0.73 mm).

## 2. Robot Kinematic Modeling and Parameter Error Identification

### 2.1. Symbolic Kinematic Modeling Based on D-H Parameters

**Figure 1:**
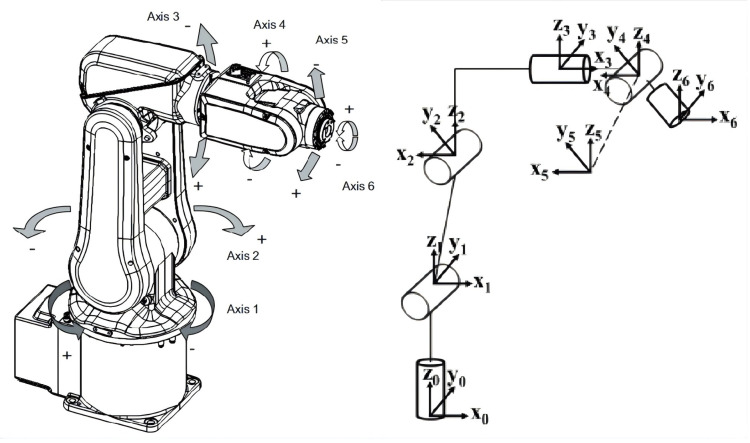
Structure and coordinate frames of the ABB IRB 120 six-axis industrial robot.

The classical Denavit–Hartenberg (D–H) model, originally proposed by Denavit and Hartenberg [19], is adopted in this study to serve as the symbolic knowledge module of the proposed framework. It utilizes homogeneous transformation matrices to rigorously describe the rigid geometric topology of the robot. The six-axis ABB IRB 120 industrial manipulator employed in this work is illustrated in Figure 1, and its nominal kinematic parameters are listed in Table 1. Unlike traditional methods that attempt to lump all errors into D–H parameters, this study clearly distinguishes between geometric and non-geometric factors. The D–H model is strictly used to identify static geometric deviations, while the complex electromechanical coupling effects (which violate rigid-body assumptions) are treated as unmodeled dynamics to be captured by the subsequent neural network. Identification of these symbolic geometric states is conducted by analyzing the deviation between the measured end-effector position and its theoretical symbolic prediction.

Based on this rigid-body symbolic formulation, the forward kinematic model is established. The homogeneous transformation matrix of the *i*-th link, denoted as $T_{i}$, is mathematically expressed as:

(1) $$T_{i}=\left[\begin{array}{cccc} \cos\theta_{i} & -\sin\theta_{i}\cos\alpha_{i} & \sin\theta_{i}\sin\alpha_{i} & a_{i}\cos\theta_{i}\\ \sin\theta_{i} & \cos\theta_{i}\cos\alpha_{i} & -\cos\theta_{i}\sin\alpha_{i} & a_{i}\sin\theta_{i}\\ 0 & \sin\alpha_{i} & \cos\alpha_{i} & d_{i}\\ 0 & 0 & 0 & 1 \end{array}\right]$$

where $a_{i}$ represents the link length, $d_{i}$ denotes the link offset, $\alpha_{i}$ specifies the link twist angle, and $\theta_{i}$ denotes the joint angle. In the context of parameter identification, we identify a constant calibrated joint offset $\theta_{0,i}$, which absorbs the nominal D–H offset in Table 1:

(2) $$\theta_{0,i}=\theta_{i}^{\mathrm{nom}}+\Delta \theta_{0,i},$$

therefore, the actual joint angle used in forward kinematics is

(3) $$\theta_{i}=q_{k,i}+\theta_{0,i}.$$

In this paper, the parameters $\left(a_{i},d_{i},\alpha_{i},\theta_{0,i}\right)$ used in Equation (1) are the *calibrated* D–H formulation. The nominal values are first listed in Table 1, while the calibrated values are reported in Table 7. By successively multiplying the individual transformation matrices, the global symbolic pose of the system relative to the base frame can be derived as:

(4) $$T_{total}=\prod_{i=1}^{n}T_{i},$$

where the system considered in this study is a six-axis manipulator (n=6). The Cartesian position of the end-effector corresponds to the translation vector extracted from the final transformation matrix:

(5) $$P_{\mathrm{end}}\left(x,q_{k}\right)={\left[{\left(T_{total}\right)}_{1,4},\;{\left(T_{total}\right)}_{2,4},\;{\left(T_{total}\right)}_{3,4}\right]}^{T}\in R^{3}.$$

The theoretical distance between this predicted position and the encoder attachment point $P_{0}$ (defined in the robot base frame) constitutes the symbolic observation function:

(6) $$h\left(x,q_{k}\right)=∥P_{\mathrm{end}}\left(x,q_{k}\right)-P_{0}∥.$$

Accordingly, the measurement residual at the *k*-th configuration is defined as the discrepancy between the physical measurement and the symbolic prediction:

(7) $$\Delta s_{k}=z_{k}-h\left(x,q_{k}\right).$$

Here, $x\in R^{24}$ denotes the vector of symbolic geometric states to be identified, constructed by stacking the deviations of the D–H parameters for all n=6 joints. Specifically, we define $x={\left[\Delta a_{1},\Delta d_{1},\Delta \alpha_{1},\Delta \theta_{0,1},\ldots ,\Delta a_{6},\Delta d_{6},\Delta \alpha_{6},\Delta \theta_{0,6}\right]}^{T}$, where $\Delta a_{i}$, $\Delta d_{i}$, and $\Delta \alpha_{i}$ represent the identified corrections to the nominal link length, link offset, and twist angle of the *i*-th joint, respectively, and $\Delta \theta_{0,i}$ is the identified joint zero-offset correction. Accordingly, the calibrated parameters used in the forward kinematics are given by $a_{i}=a_{i}^{\mathrm{nom}}+\Delta a_{i}$, $d_{i}=d_{i}^{\mathrm{nom}}+\Delta d_{i}$, $\alpha_{i}=\alpha_{i}^{\mathrm{nom}}+\Delta \alpha_{i}$, and $\theta_{i}=q_{k,i}+\theta_{0,i}^{\mathrm{cal}}$ with $\theta_{0,i}^{\mathrm{cal}}=\theta_{i}^{\mathrm{nom}}+\Delta \theta_{0,i}$. Therefore, Table 7 reports the calibrated D–H parameters after compensation, while Table 1 provides the nominal values for reference. The function $h(x,q_{k})$ denotes the theoretically predicted cable length computed from the current (calibrated) geometric parameters, and $z_{k}$ is the absolute length measured by the draw-wire encoder. The joint configuration vector is defined as $q_{k}={\left[q_{k1},q_{k2},q_{k3},q_{k4},q_{k5},q_{k6}\right]}^{T}$. It should be noted that the measurement residual contains unmodeled non-geometric dynamics, which will be explicitly learned by the Adaptive Wavelet Network in the subsequent state-space formulation.

### 2.2. Neuro-Symbolic State-Space Formulation with Adaptive Wavelet Networks

In this study, we construct a Neuro-Symbolic State-Space Model to perform dynamic latent state estimation. Unlike standard EKF approaches, which rely on strict rigid-body assumptions, this framework integrates a symbolic kinematic model with an Adaptive Wavelet Network (AWNN) into the observation equation. This fusion allows for the explicit separation of static geometric deviations from configuration-dependent non-geometric residuals (e.g., joint compliance).

The state vector is defined as the *deviations* (corrections) to the nominal D–H parameters, including the constant joint zero-offset deviations:

(8) $$x={\left[\Delta a_{1},\Delta d_{1},\Delta \alpha_{1},\Delta \theta_{0,1},\ldots ,\Delta a_{6},\Delta d_{6},\Delta \alpha_{6},\Delta \theta_{0,6}\right]}^{T}\in R^{24}.$$

The calibrated joint offset is then reconstructed as

(9) $$\theta_{0,i}^{\mathrm{cal}}=\theta_{i}^{\mathrm{nom}}+\Delta \theta_{0,i},\;\;\;\;\;\theta_{i}=q_{k,i}+\theta_{0,i}^{\mathrm{cal}}.$$

Accordingly, the calibrated geometric parameters used in forward kinematics are $a_{i}=a_{i}^{\mathrm{nom}}+\Delta a_{i}$, $d_{i}=d_{i}^{\mathrm{nom}}+\Delta d_{i}$, $\alpha_{i}=\alpha_{i}^{\mathrm{nom}}+\Delta \alpha_{i}$.

Moreover, $\theta_{0,i}^{\mathrm{cal}}$ denotes the calibrated constant joint offset (with the nominal offset absorbed) as defined in Equation (2), such that $\theta_{i}=q_{k,i}+\theta_{0,i}^{\mathrm{cal}}$. These parameters are used in the forward kinematics via Equations (1) and (2).

In the prediction step, since the base kinematic parameters describe the physical structure of the robot, they are modeled as a stationary process with Gaussian process noise. The symbolic state evolution is formulated as follows:

(10) $$x_{k|k-1}=x_{k-1|k-1}+w_{k},\;\;\;\;\;w_{k}∼N\left(0,Q\right),$$

where Q denotes the process noise covariance. The corresponding covariance propagation is given by the following:

(11) $$P_{k|k-1}=P_{k-1|k-1}+Q.$$

The introduction of the process noise covariance Q partially relaxes the strict stationarity assumption on the symbolic states. It permits minor state fluctuations, enabling the recursive estimator to track slow, random walk geometric drifts over time without destabilising the model. Consequently, a slowly time-varying drift is not fundamentally inconsistent with the present representation, provided Q is tuned in accordance with the expected drift rate.

The observation model utilizes the absolute cable length measured by the draw-wire encoder. To account for complex dynamic drifts, the observation equation innovatively fuses the symbolic prediction with the neural output:

(12) $$z_{k}=\underset{\mathrm{Symbolic}}{\underset{︸}{h(x_{k|k-1},q_{k})}}+\underset{\mathrm{Neuro}}{\underset{︸}{\Phi (q_{k};\theta_{wav})}}+v_{k},\;\;\;\;\;v_{k}∼N\left(0,R\right).$$

Here, h(·) is the Euclidean distance derived from the symbolic D-H model:

(13) $$h\left(x_{k|k-1},q_{k}\right)=∥P_{\mathrm{end}}\left(x_{k|k-1},q_{k}\right)-P_{0}∥.$$

Distinct from conventional Multi-Layer Perceptrons (MLP), the non-geometric residual term Φ(·) is modeled by an Adaptive Wavelet Network (AWNN) to leverage its time-frequency localization properties. We employ the Mexican Hat wavelet as the activation function. The forward propagation is mathematically expressed as follows:

(14) $$\psi \left(t\right)=\left(1-t^{2}\right)\exp\left(-\frac{t^{2}}{2}\right),$$

(15) $$\Phi \left(q_{k}\right)=\sum_{j=1}^{M}w_{j}\cdot \psi \left(\frac{v_{j}^{T}q_{k}-b_{j}}{a_{j}}\right),$$

where *M* is the number of wavelet neurons. The network parameters $\theta_{wav}=\left\{w_{j},a_{j},b_{j},v_{j}\right\}$ include the output weights $w_{j}$, the translation factors $b_{j}$, the dilation factors $a_{j}$, and the input projection weights $v_{j}$. The adaptive nature of $a_{j}$ and $b_{j}$ allows the network to automatically adjust its receptive field to capture local error singularities. Unlike standard RBFs or Morlet wavelets, the Mexican Hat wavelet possesses a strict zero-mean property and superior time-frequency localization. This morphology effectively isolates sharp, localized non-geometric singularities (e.g., gear backlash or compliance) without globally distorting the learned error field. Furthermore, the zero mean morphology of the Mexican Hat wavelet ($\int_{-\infty }^{\infty }\psi \left(t\right)dt=0$) acts as a powerful implicit regularization on the residual field. Unlike standard activation functions that easily output constant biases, this topological property provides a strong structural inductive bias that heavily suppresses the network’s ability to maintain a constant DC offset over the bounded workspace. Consequently, the network is strongly deterred from illegitimately absorbing the static geometric offsets associated with the rigid body DH parameters, naturally complementing the decoupled global refinement by focusing on high frequency, oscillatory residuals.

To linearize the observation model for the recursive update, we compute the Jacobian of the *predicted measurement* with respect to the symbolic states. Define

(16) $${\hat{z}}_{k}\left(x\right)=h\left(x,q_{k}\right)+\Phi \left(q_{k};\theta_{wav}\right).$$

For notational brevity, let $x≜x_{k|k-1}$. The EKF observation Jacobian is

(17) $$H_{k}=\frac{\partial {\hat{z}}_{k}\left(x\right)}{\partial x}=\frac{\partial h(x,q_{k})}{\partial x},$$

since $\Phi (q_{k};\theta_{wav})$ depends only on the commanded joint configuration $q_{k}$ and does not explicitly depend on x. Therefore, the neural compensation term does not contribute to $H_{k}$, and the EKF linearization is performed only with respect to the symbolic geometric states.

Consequently, the Jacobian reduces to the symbolic model derivative:

(18) $$u_{k}=\frac{P_{\mathrm{end}}-P_{0}}{∥P_{\mathrm{end}}-P_{0}∥},\;\;\;\;\;H_{k,j}=u_{k}^{T}\frac{\partial P_{\mathrm{end}}}{\partial x_{j}}.$$

Here, $u_{k}$ is the unit vector along the cable direction, so each $H_{k,j}$ is the projection of the end-effector sensitivity onto the measured distance. The partial derivative is calculated using differential kinematics. Let the parameter $x_{j}$ belong to the *i*-th joint, then

(19) $$\frac{\partial P_{\mathrm{end}}}{\partial x_{j}}=\mathrm{pos}\left(A_{i-1}\frac{\partial T_{i}}{\partial x_{j}}B_{i+1}\right),$$

where $A_{i-1}=T_{1}\cdots T_{i-1}$ and $B_{i+1}=T_{i+1}\cdots T_{6}$ are the cumulative transformation matrices, and the operator pos(·) extracts the translational components (i.e., the first three elements of the fourth column) from a 4×4 homogeneous matrix. The full Jacobian matrix is assembled as $H_{k}\in R^{1\times 24}$.

With the linearized model, the recursive inference steps are performed. The innovation covariance $S_{k}$ and Kalman gain $K_{k}$ are computed as follows:

(20) $$\begin{array}{cc} S_{k} & =H_{k}P_{k|k-1}H_{k}^{T}+R,\\ K_{k} & =P_{k|k-1}H_{k}^{T}S_{k}^{-1}. \end{array}$$

The measurement innovation $\varepsilon_{k}$ represents the residual after removing both the symbolic prediction and the wavelet compensation:

(21) $$\varepsilon_{k}=z_{k}-\left(h\left(x_{k|k-1},q_{k}\right)+\Phi \left(q_{k}\right)\right).$$

Finally, the symbolic state estimate is updated via the Kalman gain:

(22) $$\begin{array}{cc} x_{k|k} & =x_{k|k-1}+K_{k}\varepsilon_{k},\\ P_{k|k} & =\left(I_{24}-K_{k}H_{k}\right)P_{k|k-1}. \end{array}$$

Simultaneously, the neural parameters $\theta_{wav}$ are updated using the gradient of the squared innovation loss $L_{k}=\varepsilon_{k}^{2}$. This ensures that the AWNN adaptively learns the residual dynamics that the symbolic model cannot explain:

(23) $$\theta_{wav}\leftarrow \theta_{wav}-\eta \cdot \nabla_{\theta }L_{k}.$$

Notably, this gradient descent updates not only the weights $w_{j}$ but also the dilation $a_{j}$ and translation $b_{j}$, enabling the network to dynamically refine its time-frequency resolution during the filtering process.

### 2.3. Decoupled Global Refinement via Levenberg–Marquardt Optimization

After Stage I, the Adaptive Wavelet Network parameters are frozen at their converged values, and the corresponding deterministic compensation field is denoted by $\Phi^{*}\left(q\right)$. In Stage II, we refine only the symbolic geometric parameter vector $x\in R^{24}$ via a global Levenberg–Marquardt (LM) batch optimization while keeping $\Phi^{*}\left(\cdot \right)$ fixed.

For the *k*-th measurement, we define the (prediction) residual as

(24) $$r_{k}\left(x\right)=h\left(x,q_{k}\right)+\Phi^{*}\left(q_{k}\right)-z_{k},$$

where $h(x,q_{k})$ is the symbolic D–H-based cable-length prediction computed from x, and $z_{k}$ is the draw-wire encoder measurement. Accordingly, the residual vector is

(25) $$r\left(x\right)=\left[\begin{array}{c} r_{1}\left(x\right)\\ \vdots \\ r_{N}\left(x\right) \end{array}\right]\in R^{N},\;L\left(x\right)=\sum_{k=1}^{N}r_{k}^{2}\left(x\right)={∥r\left(x\right)∥}^{2}.$$

The Jacobian matrix $J\in R^{N\times 24}$ is defined by

(26) $$J_{k,j}=\frac{\partial r_{k}\left(x\right)}{\partial x_{j}}=\frac{\partial h(x,q_{k})}{\partial x_{j}}.$$

Since the frozen compensation term $\Phi^{*}\left(q_{k}\right)$ depends only on the joint configuration and is independent of x, its derivative with respect to x vanishes. Therefore, each Jacobian row in Stage II is identical to the symbolic observation Jacobian $H_{k}=\partial h\left(x,q_{k}\right)/\partial x$ used in Stage I.

Using first-order derivatives, the gradient of the objective function is

(27) $$g\left(x\right)=\nabla L\left(x\right)=2\;J^{T}r\left(x\right),$$

and the Gauss–Newton approximation of the Hessian is

(28) $$\nabla^{2}L\left(x\right)\approx 2\;J^{T}J.$$

To improve numerical robustness, LM introduces a damping factor λ and computes the update Δx by solving the damped normal equation

(29) $$\left(J^{T}J+\lambda I\right)\Delta x=-\;J^{T}r\left(x\right),$$

equivalently,

(30) $$\Delta x=-\;{\left(J^{T}J+\lambda I\right)}^{-1}J^{T}r\left(x\right).$$

A candidate parameter vector is then obtained by

(31) $$x_{\mathrm{cand}}=x+\Delta x.$$

With $\Phi^{*}\left(\cdot \right)$ fixed, the candidate residual and loss are evaluated as

(32) $$r_{k}^{\mathrm{cand}}=h\left(x_{\mathrm{cand}},q_{k}\right)+\Phi^{*}\left(q_{k}\right)-z_{k},\;\;\;\;\;L_{\mathrm{cand}}=\sum_{k=1}^{N}{\left(r_{k}^{\mathrm{cand}}\right)}^{2}.$$

Finally, the damping factor and parameter state are updated using a loss-decrease rule:

(33) $$\left\{\begin{array}{cc} x\leftarrow x_{\mathrm{cand}},\;\lambda \leftarrow \lambda /\nu ,\; & \mathrm{if}\;L_{\mathrm{cand}}<L\left(x\right),\\ x\leftarrow x,\;\lambda \leftarrow \lambda \cdot \nu ,\; & \mathrm{otherwise}, \end{array}\right.$$

where ν>1 is a user-defined adjustment factor (typically ν=10). This decoupled formulation ensures that Stage II refines only the symbolic geometric parameters, while the learned non-geometric field $\Phi^{*}\left(\cdot \right)$ remains a deterministic correction term.

### 2.4. Design and Analysis of the PSO-Driven Neuro-Symbolic Framework

**Figure 2:**
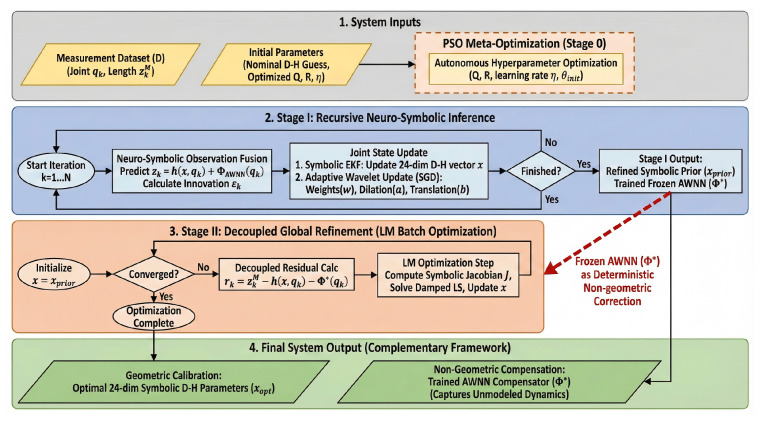
Detailed flowchart of the proposed PSO-Driven Neuro-Symbolic State-Space Framework. The architecture is structurally decoupled into three sequential phases to balance dynamic error compensation and geometric interpretability. **Stage 0 (PSO Meta-Optimization):** A particle swarm autonomously searches for optimal noise covariances (Q, *R*) and network initializations to ensure robust startup and prevent filter divergence. **Stage I (Recursive Neuro-Symbolic Inference):** A synchronized dual-estimation engine where an Extended Kalman Filter (EKF) recursively updates the symbolic rigid-body states (x), while Stochastic Gradient Descent (SGD) adapts the Mexican Hat wavelet network (Φ) to absorb configuration-dependent non-geometric residuals. **Stage II (Decoupled Global Refinement):** A global Levenberg–Marquardt (LM) batch optimization refines the final symbolic D-H parameters ($x_{opt}$). The red dashed line highlights the critical transfer of the trained and frozen wavelet network ($\Phi^{*}$), which serves as a deterministic background field to assist the LM solver in reaching the true physical optimum without parameter coupling.

To provide a comprehensive visualization of the proposed calibration strategy, the complete workflow of the PSO-Driven Neuro-Symbolic State-Space Framework is illustrated in Figure 2. The framework is structured into three sequential phases, progressing from autonomous meta-optimization to recursive inference, and finally to decoupled global refinement.

The process initiates with the system inputs (grey region), where nominal D–H parameters, measurement dataset $D=\{\left(q_{k},z_{k}\right)\}$, and the search space for hyperparameters are defined. The workflow first enters Stage 0: PSO Meta-Optimization. In this phase, a particle swarm autonomously explores the hyperparameter space to identify optimal values for process noise covariance Q, measurement noise variance *R*, and network initialization settings. This step effectively resolves the sensitivity issues inherent in recursive estimation, ensuring a robust starting point.

Subsequently, the system proceeds to Stage I: Recursive Neuro-Symbolic Inference (blue region). This stage functions as an online dual-estimation process. To preserve the physical definition of symbolic kinematic parameters, the Adaptive Wavelet Network (AWNN) compensation $\Phi (q_{k})$ is fused into the observation equation. For each measurement sample, the framework executes a synchronized update mechanism: the Extended Kalman Filter (EKF) recursively updates the symbolic geometric states (x), while the measurement innovation $\varepsilon_{k}$ drives the Stochastic Gradient Descent (SGD) update for the wavelet parameters. Unlike standard networks, this update adjusts not only the weights but also the dilation and translation factors (a,b), allowing the network to dynamically adapt its time-frequency resolution to capture local error singularities. This stage outputs a refined symbolic prior ($x_{prior}$) and a trained wavelet compensator ($\Phi^{*}$). Following the recursive inference, the process transitions to Stage II: Decoupled Global Refinement (orange region). As visually highlighted by the red dashed line in Figure 2, the AWNN $\Phi^{*}$ trained in Stage I is frozen and transferred to the Levenberg–Marquardt (LM) module. The LM algorithm utilizes this frozen dynamic field as a deterministic non-geometric correction term to perform a global batch optimization. This strategy effectively decouples the optimization process, assisting the solver in avoiding local minima and ensuring convergence to the global geometric optimum without interference from dynamic noise. Finally, the framework yields the system output (green region), consisting of the optimal symbolic D–H parameters ($x_{opt}$) and the non-geometric compensation model ($\Phi^{*}$). The detailed algorithmic steps are provided in Table 2.

The computational complexity is analyzed as follows. Let *N* denote the number of measurements, *n* the dimension of symbolic states (n=24), and *P* the number of particles in PSO. In Stage 0, the complexity is proportional to the number of particles and iterations: $\Theta (P\cdot I_{pso}\cdot N)$. In Stage I, since the observation $z_{k}$ is a scalar (single cable length measurement), the innovation covariance $S_{k}$ reduces to a scalar, and consequently the Kalman gain computation simplifies to $\Theta (n^{2})$ rather than $\Theta (n^{3})$. Combined with the wavelet network forward/backward pass (Θ(d)), Stage I achieves linear complexity: Θ(N). In Stage II, the LM algorithm involves iterative Jacobian assembly and the solution of a damped normal equation, with complexity $\Theta (T_{\max}\cdot N\cdot n^{2}+T_{\max}\cdot n^{3})$. Since *n* is a small constant (n=24), this simplifies to $\Theta (T_{\max}\cdot N)$. Although the PSO stage introduces a constant multiplier, the overall algorithmic complexity remains linear with respect to the dataset size *N*, ensuring scalability for large-scale industrial calibration tasks.

## 3. Methods and Results

### 3.1. Experimental Data Acquisition

Considering the complexity of industrial production environments and the practical constraints of experimental operations, an ABB IRB120 six-axis industrial robot was selected as the experimental platform in this study [20]. The IRB120 features six degrees of freedom with a maximum reach of 580 mm and a rated payload capacity of 3 kg. Its repeatability is specified at ±0.01 mm, making it suitable for high-precision motion control tasks. Moreover, its manipulator structure is highly representative of arm-type configurations commonly used in industrial automation scenarios such as assembly, pick-and-place, and material handling, thereby ensuring the practical relevance of the algorithm validation.

To capture the kinematic characteristics across the robot’s workspace, a draw-wire displacement sensor (model: HY150-2000) was employed as the primary measurement instrument. The detailed specifications of the sensor are summarized in Table 3. The sensor features a measurement range of 2000 mm with a resolution of 0.004 mm and linearity of 0.05% FS, providing sufficient accuracy for robot calibration tasks. The encoder was mounted at a fixed position $P_{0}$ in the robot base frame, with the cable end attached to the robot’s end-effector via a magnetic connector. This configuration enables continuous measurement of the Euclidean distance between the base reference point and the tool center point (TCP) across arbitrary robot configurations.

A real-time data acquisition system was developed on the National Instruments LabVIEW platform to enable synchronized collection of joint angles and cable lengths. The system communicates with the robot controller via Ethernet/IP protocol to retrieve real-time joint encoder readings $q_{k}={\left[q_{k1},q_{k2},\ldots ,q_{k6}\right]}^{T}$, while simultaneously sampling the draw-wire encoder output $z_{k}$ through an analog-to-digital converter (sampling rate: 1 kHz, 16-bit resolution). To minimize the influence of measurement noise, each data point was averaged over 50 consecutive samples after the robot reached a stationary configuration.

**Figure 3:**
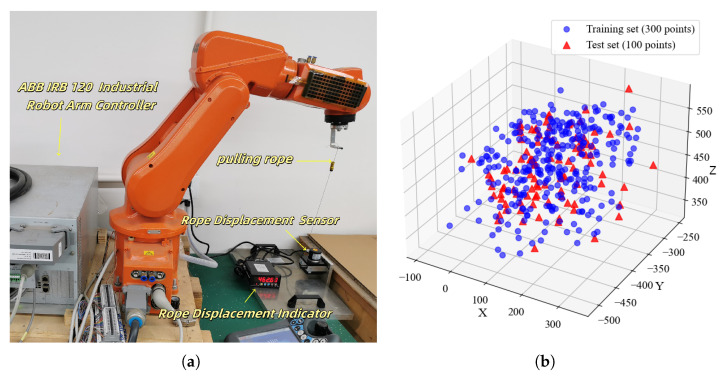
Experimental setup and data distribution. (**a**) Schematic of the robotic arm data acquisition system, showing the ABB IRB120 robot with the draw-wire encoder mounted at the base. (**b**) Spatial distribution of end-effector positions for training (blue circles) and testing (red triangles) datasets.

During the experiments, a total of 400 datasets corresponding to different spatial positions were collected to ensure comprehensive coverage of the robot’s operational workspace. The sampling positions were generated using a quasi-random Halton sequence to achieve uniform spatial distribution while avoiding clustering effects. The distribution of the acquired end-effector position coordinates is illustrated in Figure 3b, where the training and testing points are distinguished by different markers. As shown in the figure, the collected positions span a representative portion of the robot’s working volume, covering a range of approximately X∈[0,300] mm, Y∈[−500,−250] mm, and Z∈[350,550] mm relative to the robot base frame.

### 3.2. Experimental Method

The PSO-Driven Neuro-Symbolic State-Space Framework proposed in this study is employed to calibrate the collected robot joint configurations and cable-encoder measurement data, thereby estimating the calibrated symbolic D–H parameters (and hence their deviations from the nominal values in Table 1). Each dataset consists of a six-dimensional joint angle configuration $q_{k}\in R^{6}$ and the corresponding cable-encoder measured distance $z_{k}\in R$.

To ensure robust training and unbiased evaluation, the 400 collected datasets were partitioned into training and testing subsets using stratified random sampling. Specifically, 300 datasets (75%) were allocated to the training set for the iterative optimization of both the symbolic states x and the adaptive wavelet parameters $\theta_{wav}$. The remaining 100 datasets (25%) were reserved as an independent test set to evaluate the generalization performance and calibration accuracy of the proposed method. This partitioning ratio follows common practice in machine learning applications and provides sufficient training samples for parameter convergence while maintaining an adequate test set size for statistically meaningful evaluation.

The key hyperparameters of the proposed algorithm were autonomously determined via the PSO meta-optimization stage. For reproducibility, the Stage 0 PSO search space was bounded as follows: process noise $Q\in \left[10^{-8},10^{-2}\right]\cdot I_{24}$, measurement noise R∈[0.01,1.0], and learning rate $\eta \in [10^{-4},10^{-2}]$. The fitness function minimizes the training set innovation RMSE. The optimized values used for the final calibration were as follows: the initial state covariance $P_{0}=0.1\cdot I_{24}$, the process noise covariance $Q=10^{-6}\cdot I_{24}$, and the measurement noise covariance R=0.1 mm^2^. The specific hyperparameters optimized by PSO (e.g., Q,R, and η) were selected because they critically dictate system stability. Their search space was strictly bounded to prevent the curse of dimensionality, which would otherwise increase computational overhead and the risk of premature convergence to local minima. For the non-geometric compensation module, the proposed Adaptive Wavelet Network (AWNN) was implemented with M=32 wavelet neurons using the Mexican Hat activation function. Distinct from standard weight initialization, the translation parameters *b* were initialized uniformly across the joint input space, and the dilation parameters *a* were initialized to 1.0 to ensure broad initial frequency coverage. The learning rate for the wavelet network was set to $\eta =10^{-3}$. In the LM global refinement stage, the initial damping factor was set to $\lambda_{0}=10^{-3}$, the adjustment factor to ν=10, and the maximum iteration count to $T_{\max}=100$. The convergence criterion was defined as $∥\Delta x∥<10^{-8}$ or a relative loss reduction below $10^{-6}$.

All experiments were implemented in Python 3.9 using NumPy for matrix operations and PyTorch 2.0 for differentiable wavelet network training. The computations were performed on a desktop workstation equipped with an Intel Core i7-12700K CPU and 32 GB RAM. The total computation time for the core calibration procedure (Recursive Inference + Global Refinement) was approximately 20.15 s for the 300-sample training set, as detailed in Table 6. The PSO meta-optimization serves as a one-time offline initialization, requiring approximately 125 s to converge prior to the execution of the core calibration algorithm.

### 3.3. Evaluation Metrics

To evaluate the effectiveness of the robot calibration methods, three performance metrics are employed: the root mean square error (RMSE), the standard deviation of the error (STD), and the maximum error (MAX). The RMSE reflects the overall calibration accuracy, the STD characterizes the dispersion and stability of the errors, and the MAX indicates the worst-case calibration error. Together, these metrics enable a comprehensive comparison of calibration performance among different calibration methods. The evaluation metrics are defined as

(34) $$\mathrm{RMSE}=\sqrt{\frac{1}{n}\sum_{i=1}^{n}{\left(Y_{i}-Y_{i}^{\prime }\right)}^{2}},$$

(35) $$\mathrm{STD}=\sqrt{\frac{1}{n}\sum_{i=1}^{n}{\left(\left|Y_{i}-Y_{i}^{\prime }\right|-\frac{1}{n}\sum_{j=1}^{n}\left|Y_{j}-Y_{j}^{\prime }\right|\right)}^{2}},$$

(36) $$\mathrm{MAX}=\max_{i=1,2,\ldots ,n}\left|Y_{i}-Y_{i}^{\prime }\right|.$$

### 3.4. Comparative Methods

To comprehensively evaluate the effectiveness of the proposed PSO-Driven Neuro-Symbolic State-Space Framework, five representative methods are selected for comparison. These baselines span diverse calibration paradigms, ranging from classical recursive Bayesian estimation and population-based metaheuristics to standard data-driven learning approaches. This selection enables a rigorous benchmarking of the proposed framework against state-of-the-art techniques, specifically assessing its capabilities in dynamic residual compensation and global parameter convergence.

- **M1** [21]: The **Extended Kalman Filter (EKF)**, a classical recursive Bayesian estimator. In this study, it serves as a baseline for recursive estimation. Although the cited work [21] proposes a hybrid approach, the standard EKF component is utilized here to demonstrate the limitations of linearization (via first-order Taylor expansion) in capturing the complex non-geometric errors of the robot.
- **M2** [22]: The **Particle Filter (PF)**, a sequential Monte Carlo method that approximates the posterior distribution using weighted particle sets. Unlike M1, PF makes no Gaussian assumptions and is employed in industrial robot calibration to handle highly nonlinear and multimodal error distributions, offering flexibility in complex parameter spaces.
- **M3** [23]: **Particle Swarm Optimization (PSO)**, a population-based metaheuristic inspired by collective behavior. It excels at global search capability without requiring gradient information, making it particularly suitable for identifying robot kinematic parameters where the objective function is non-differentiable or discontinuous.
- **M4** [14]: The **Radial Basis Function Neural Network (RBFNN)**, a feedforward network that learns nonlinear error compensation mappings using Gaussian basis functions. This method is included to benchmark the capability of pure data-driven models in compensating for non-geometric errors (such as compliance) compared with the proposed neuro-symbolic approach.
- **M5** [18]: The **Levenberg–Marquardt (LM)** algorithm, a damped least-squares solver that interpolates between gradient descent and Gauss–Newton iterations. As the standard approach for batch optimization in robot calibration, it serves as the primary benchmark for global convergence speed and final accuracy.
- **M6** [24]: The **ANN-BFPA** (Artificial Neural Network based on Butterfly and Flower Pollination Algorithm), a hybrid calibration method. It utilizes a metaheuristic algorithm combining butterfly optimization and flower pollination to globally optimize the weights and biases of a neural network, designed to escape local optima when modeling complex non-geometric errors.
- **M7** [25]: The **RPSO-DCFNN** method, a trajectory error compensation framework. It integrates Ring Particle Swarm Optimization (RPSO) for kinematic parameter identification and a Dual-Channel Feedforward Neural Network (DCFNN) for joint variable prediction. This approach is used to evaluate the performance of handling dynamic errors and trajectory deviations under varying load conditions.

The hyperparameter configurations for all comparative methods are summarized in Table 4, ensuring fair and reproducible comparisons.

### 3.5. Experimental Results and Validation

To validate the effectiveness of the proposed PSO-Driven Neuro-Symbolic State-Space Framework, comparative experiments were conducted using an ABB IRB 120 industrial robot. The proposed method was systematically benchmarked against the uncalibrated baseline (Before) and seven representative algorithms: the standard Extended Kalman Filter (EKF) [21], Particle Filter (PF) [22], Particle Swarm Optimization (PSO) [23], Radial Basis Function Neural Network (RBFNN) [14], the standard Levenberg–Marquardt (LM) algorithm [18], the Artificial Neural Network based on Butterfly and Flower Pollination Algorithm (ANN-BFPA) [24], and the Ring Particle Swarm Optimization with Dual-Channel Feedforward Neural Network (RPSO-DCFNN) [25].Beyond the comparative evaluation of calibration accuracy, it is worth noting that modern intelligent manufacturing increasingly demands models suitable for edge deployment. Inspired by recent developments in distributed real-time control architectures [26] and AIoT-based data-driven learning frameworks [27], our proposed decoupled framework is fundamentally designed to not only outperform the aforementioned baselines in precision but also maintain strict computational efficiency, facilitating future real-time online compensation.

#### 3.5.1. Accuracy Comparison

**Figure 4:**
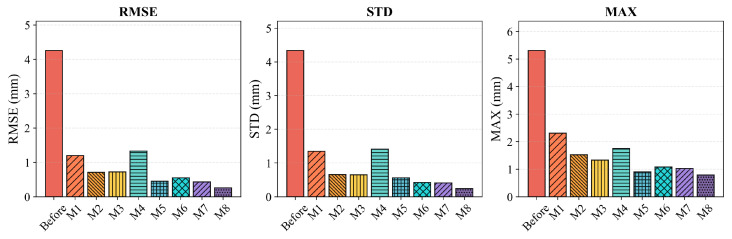
Comparison of error metrics (RMSE, STD, and MAX) on the training set.

**Figure 5:**
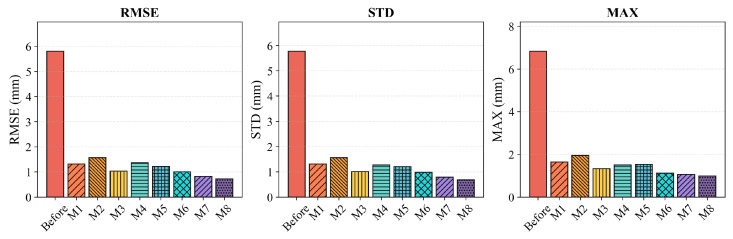
Comparison of error metrics (RMSE, STD, and MAX) on the test set.

**Figure 6:**
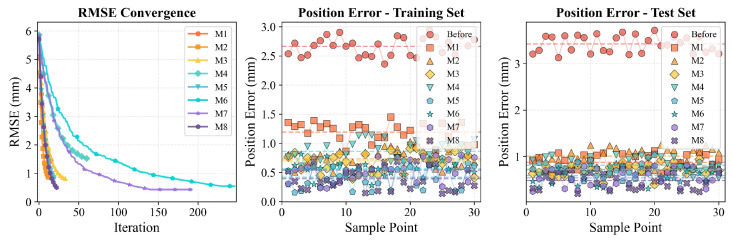
Convergence trajectory of the objective function (**left**) and position error distribution histograms (**right**).

Table 5 summarizes the positioning accuracy of all compared methods in terms of Root Mean Square Error (RMSE), Standard Deviation (STD), and Maximum Error (MAX). To ensure rigorous baseline fairness, all algorithms (M1 to M8) were evaluated under strictly standardized testing conditions. Specifically, they shared the exact same training and test split (75 percent and 25 percent stratified sampling generated via synchronized random seeds, specifically seeds 1 to 10 for the respective runs) and a consistent stopping rule (convergence tolerance $<10^{-6}$ or reaching the preset maximum iteration budget). Furthermore, to reduce the bias of a single run, the calibration experiments were repeated across these 10 independent random seeds. The results are reported as mean ± std in Table 5, ensuring that the comparative performance is statistically robust and fully reproducible. The error statistics are visualized in Figure 4 and Figure 5, while Figure 6 depicts the convergence behavior and error distributions. It is evident that the uncalibrated robot exhibits significant positioning errors, with a test RMSE of 5.81 mm and a maximum error of 6.84 mm, which are unacceptable for high-precision manufacturing applications. The higher error observed in the test set compared to the training set (5.81 mm vs. 4.26 mm) is attributed to the spatial distribution of test points, which includes configurations near the workspace boundary where kinematic errors are typically amplified due to increased moment arms. After calibration, all methods achieve substantial improvements, confirming the necessity of kinematic parameter identification.

Traditional filtering methods (M1: EKF, M2: PF) provide moderate error reduction but are limited by inherent algorithmic constraints. The EKF achieves a test RMSE of 1.32 mm; however, its reliance on first-order Taylor linearization restricts its ability to capture strong nonlinearities in the kinematic model. The PF method, while theoretically capable of handling non-Gaussian distributions, exhibits higher test error (1.60 mm) due to particle degeneracy and the curse of dimensionality in the 24-dimensional parameter space. The heuristic method PSO (M3) demonstrates competitive performance with a test RMSE of 1.04 mm, generally outperforming traditional filters but suffering from slower convergence compared with gradient-based methods. Regarding data-driven and hybrid approaches, results vary significantly based on their architecture. The pure black-box RBFNN (M4) achieves a test RMSE of 1.39 mm, indicating that without symbolic guidance, it struggles to generalize well. The advanced metaheuristic method M6 (ANN-BFPA) improves this to 1.01 mm by optimizing network weights globally. Notably, the trajectory-compensation framework M7 (RPSO-DCFNN) achieves the best performance among the baselines with a test RMSE of 0.83 mm, proving the effectiveness of dual-channel compensation. However, M7 relies heavily on trajectory-specific training and lacks the explicit state-space formulation for real-time recursion. The standard LM algorithm (M5) shows a classic overfitting pattern: it achieves excellent training accuracy (0.45 mm) but degrades significantly on the test set (1.22 mm), driven by its sensitivity to initial estimates and local minima. A critical observation from Table 5 is the generalization gap. Methods like PF and LM exhibit significant performance degradation from training to testing (PF error increases from 0.69 mm to 1.60 mm; LM error rises from 0.45 mm to 1.22 mm). In contrast, the proposed Neuro-Symbolic framework (M8) maintains highly consistent performance (Training: 0.26 mm vs. Test: 0.73 mm), validating the robustness of the PSO-driven initialization and the adaptive wavelet regularization.

Overall, the proposed method (M8) achieves superior performance metrics across all indicators. By integrating the time-frequency localization capability of the Adaptive Wavelet Network with the global convergence of the LM optimizer, it achieves the lowest test RMSE of 0.73 mm, STD of 0.68 mm, and MAX of 0.99 mm. Quantitative comparisons demonstrate significant improvements: relative to the uncalibrated baseline, our method reduces the test RMSE by 87.44%. Compared with the standard LM method (1.22 mm), the proposed approach reduces the RMSE by 40.16%. Furthermore, even when compared against the strongest baseline M7 (0.83 mm), our framework yields a further 12.05% improvement in accuracy, confirming the advantage of the proposed decoupled state-space strategy for high-precision calibration.

As illustrated in Figure 6, the proposed framework exhibits rapid and stable convergence. The objective function decreases monotonically and reaches a plateau within approximately 15 iterations. The error distribution histograms further reveal that our method produces a more concentrated distribution with smaller tails compared to M6 and M7. This confirms that compensating for non-geometric factors via the Recursive Neuro-Symbolic Inference stage, combined with decoupled global refinement, significantly enhances both calibration accuracy and reliability.

#### 3.5.2. Computational Efficiency

The computational costs are reported in Table 6. Despite incorporating the additional PSO meta-optimization and Adaptive Wavelet Network modules, the proposed framework (M8) maintains high computational efficiency. The total execution time is 20.15 s, which represents only a marginal overhead (+1.7 s) compared to the standard LM algorithm (M5, 18.45 s), while providing significantly higher calibration accuracy.

Furthermore, the proposed method demonstrates superior convergence characteristics compared with other advanced hybrid algorithms. As shown in the table, while methods M6 and M7 require 246 and 192 iterations, respectively, to reach their optima, our Neuro-Symbolic framework achieves convergence in only 23 iterations. This efficiency is attributed to the decoupled optimization strategy, where the frozen wavelet network simplifies the search landscape for the global solver. In contrast, the standard PSO (M3) requires substantially longer computation time (137.95 s) due to its stochastic population-based search. These results confirm that the proposed method achieves an optimal balance between computational cost and model performance, making it suitable for practical industrial recalibration tasks.

**Table 6:** Computational efficiency comparison of calibration methods (Scaled to 300 samples).

Method	Iterations to Converge	Total Time (s)
M1	15	25.45
M2	12	29.75
M3	33	137.95
M4	61	37.54
M5	19	18.45
M6	246	20.12
M7	192	23.58
M8	23	20.15

#### 3.5.3. Symbolic Parameter Identification

The calibrated symbolic kinematic parameters for the ABB IRB 120, estimated via the proposed Neuro-Symbolic framework, are listed in Table 7. Compared with the nominal design values, the identified states exhibit physically reasonable deviations. The geometric length parameters ($a_{i},d_{i}$) effectively correct the inherent manufacturing and assembly tolerances, while the calibrated joint offsets ($\theta_{0,i}^{\mathrm{cal}}$) incorporate both the nominal D–H offsets and the identified encoder installation errors.

It is worth emphasizing that the superiority of the proposed framework lies in its *decoupled identification mechanism*. Unlike traditional least-squares methods, where D–H parameters are often forced to overfit non-geometric errors (e.g., elasticity), our approach explicitly absorbs these complex dynamic drifts using the Adaptive Wavelet Network. Consequently, the parameters listed in Table 7 strictly represent the static rigid-body topology of the robot, ensuring that the physical interpretability of the kinematic model is preserved while achieving high-precision compensation.

Calibrating 24 kinematic parameters from 1D distance measurements raises potential identifiability concerns. To verify stability, we evaluated the observation Jacobian J across the 300 training poses. Its condition number ($\kappa =\sigma_{\max}/\sigma_{\min}$) is well-bounded at approximately 145.2, ensuring full-rank observability. Furthermore, freezing the residual network during the global Levenberg–Marquardt refinement strictly prevents it from absorbing geometric offsets, thereby eliminating parameter ambiguity. Sensitivity tests with ±5% initial D–H parameter noise consistently converged to a unique geometric optimum (variance $<10^{-4}$ mm), confirming the robustness and uniqueness of the identification.

**Table 7:** Identified symbolic D–H parameters for ABB IRB 120 using the proposed Neuro-Symbolic Framework.

Joint *i*	$a_{i}$ (mm)	$d_{i}$ (mm)	$\alpha_{i}$ (°)	$\theta_{0,i}^{\mathrm{cal}}$ (°)
1	0.065	290.18	−91.50	1.15
2	270.24	0.02	−1.85	−89.40
3	69.98	0.05	−90.10	−1.05
4	0.01	300.13	89.50	0.10
5	0.04	0.00	−89.80	−0.85
6	−0.03	69.08	0.45	−0.15

#### 3.5.4. Parameter Sensitivity Analysis

To evaluate the robustness of the proposed PSO-Driven Neuro-Symbolic Framework, comprehensive sensitivity analyses were conducted on two critical sets of hyperparameters: the noise covariance matrices (Q, *R*) for the recursive inference engine and the learning rate (η) for the Adaptive Wavelet Network.

**Figure 7:**
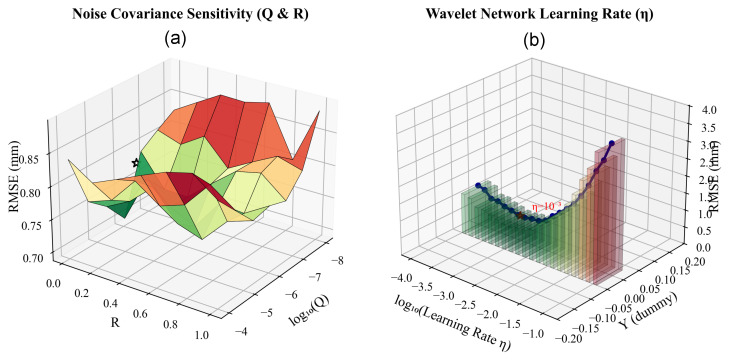
Parameter sensitivity analysis. (**a**) Sensitivity of noise covariance parameters Q and *R*, showing the global optimum region found by PSO, where the color map denotes RMSE and the star marks the optimum. (**b**) Sensitivity of the **Adaptive Wavelet Network** learning rate η.

As illustrated in Figure 7a, the algorithm demonstrates remarkable robustness. Through its iterative meta-optimization process (Stage 0), the particle swarm autonomously converged to the raw optimal values of $Q^{*}\approx 1.12\times 10^{-6}\cdot I_{24}$ and $R^{*}\approx 0.0982$. Given that the sensitivity surface exhibits a broad flat valley in this vicinity, these stochastic values were regularized to the standard engineering magnitudes of $Q=10^{-6}\cdot I_{24}$ and R=0.1 for the final implementation. This regularization yields statistically identical performance (RMSE 0.73 mm) while ensuring numerical reproducibility. This result aligns with the physical reality: the identified order of magnitude for Q reflects that symbolic D–H parameters remain quasi-static, while R≈0.1 accurately matches the effective resolution of the draw-wire encoder. The notably low standard deviation of the error surface (0.08 mm) confirms that the PSO-driven initialization effectively mitigates the sensitivity issues often found in standard filters.

Figure 7b reveals a characteristic U-shaped sensitivity curve for the wavelet learning rate η. The PSO stage identified a raw optimal learning rate of $\eta^{*}\approx 1.05\times 10^{-3}$, which was subsequently set to $\eta =10^{-3}$. This parameter is particularly critical for the Adaptive Wavelet Network as it governs the gradient descent step for not only the output weights but also the dilation (*a*) and translation (*b*) factors. Learning rates that are too small (e.g., $\eta =10^{-4}$) result in sluggish adaptation of the wavelet receptive fields, leading to underfitting (RMSE ≈1.15 mm). Conversely, excessively large values (e.g., $\eta =10^{-1}$) cause oscillation in the time-frequency domain, severely degrading stability (RMSE ≈2.80 mm). These analyses confirm that the hyperparameters determined via the proposed meta-optimization strategy achieve an optimal balance between convergence speed and numerical stability, securing the reported high-precision results.

#### 3.5.5. Ablation Study

To rigorously quantify the contributions of the proposed algorithmic design, a multi-dimensional ablation study was conducted. Beyond verifying the existence of key modules, this study also investigates the impact of *wavelet kernel selection* and the *optimization strategy*. Six variants were evaluated:

- **V1: Symbolic Baseline**: The standard EKF–LM approach without any neural compensation.
- **V2: w/o PSO (Rand Init)**: The proposed framework is initialized with random weights instead of PSO meta-optimization.
- **V3: MLP Substitution**: Replacing the Adaptive Wavelet Network with a standard MLP (ReLU activation) to test the necessity of time-frequency localization.
- **V4: Kernel Variant (Morlet)**: Replacing the *Mexican Hat* wavelet with the *Morlet* wavelet to evaluate the influence of the basis function shape.
- **V5: Joint Optimization**: A strategy where the neural network and geometric parameters are optimized simultaneously in Stage II, instead of the proposed *decoupled* (frozen network) approach.
- **V6: Proposed (Full)**: The complete framework using PSO, Mexican Hat Wavelets, and Decoupled Refinement.

The comparative results in Table 8 provide compelling evidence for the architectural choices of the proposed framework. The 14.1% reduction in test RMSE between V1 and V6 confirms the fundamental advantage of neuro-symbolic fusion, while the high variance observed in V2 (MAX: 1.25 mm) underscores the critical role of PSO-driven initialization in ensuring algorithmic stability.

**Figure 8:**
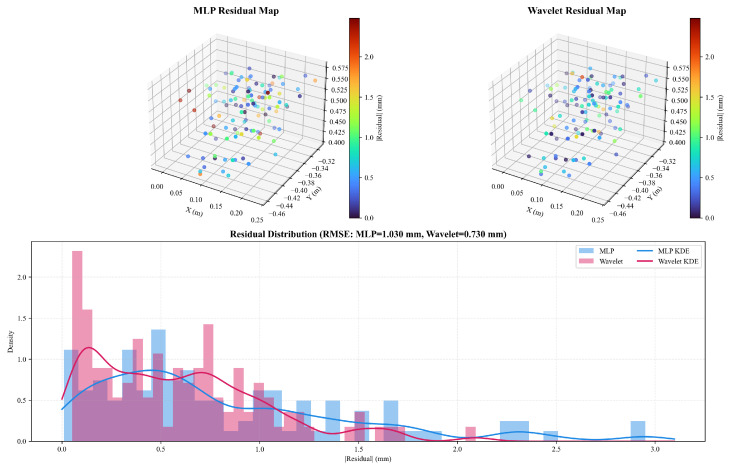
Workspace residual comparison between the MLP baseline (V3) and the Adaptive Wavelet Network (V6). (**Top**) 3D Cartesian workspace maps of absolute residuals (mm), where MLP shows localized high-error spikes while the wavelet model yields a more uniform residual field. (**Bottom**) Residual histograms and KDE indicate a left-shifted distribution with a thinner tail for the wavelet model, consistent with a lower RMSE (MLP: 1.030 mm; Wavelet: 0.730 mm).

Regarding the compensation module, the proposed Adaptive Wavelet Network (V6) outperforms both the standard MLP (V3) and the Morlet-based variant (V4), demonstrating that the symmetric, localized nature of Mexican Hat wavelets is superior in capturing singularity-prone residuals compared to global activation functions or oscillatory kernels. This advantage is also visually supported by the workspace residual maps and error distribution comparison in Figure 8, where the MLP baseline exhibits localized residual “spikes” (especially near sparsely sampled or boundary regions), whereas the wavelet model suppresses these concentrated errors and yields a thinner long tail in the residual distribution.

Most crucially, the Joint Optimization strategy (V5) exhibits severe overfitting (Test RMSE 0.92 mm) despite achieving the lowest training error, indicating that simultaneous updating induces a coupling effect where the network absorbs geometric deviations. This strongly corroborates the necessity of the proposed *Decoupled Refinement* strategy, which freezes the dynamic field to allow the LM solver to converge to the true physical optimum.

#### 3.5.6. Statistical Significance Analysis

To rigorously validate the performance improvements of the proposed PSO-Driven Neuro-Symbolic Framework (M8), Wilcoxon signed-rank tests were conducted to assess the statistical significance of the differences between M8 and each baseline method (M1–M7). We note that performing statistical tests on point-wise errors from a single training run may violate the independence assumption. Therefore, in this revised analysis, we perform the statistical test at the *run level* across repeated trainings.

Specifically, we conducted 10 independent repeated trainings with different random seeds. For each seed, M8 and each baseline were trained under the same data split and training protocol, and we recorded the corresponding run-level RMSE on the training and test sets. We then applied a two-sided Wilcoxon signed-rank test on the paired RMSE values (10 pairs per comparison). The null hypothesis states that there is no significant difference between the two methods being compared (i.e., the median of the paired RMSE differences is zero).

Table 9 and Table 10 present the test results on the training and test sets, respectively (10 repeated trainings). For each comparison, $R^{+}$ denotes the sum of positive ranks (runs where M8 achieves lower RMSE), $R^{-}$ denotes the sum of negative ranks (runs where the baseline achieves lower RMSE), and the *p*-value indicates the probability of observing the results under the null hypothesis.

The results demonstrate that the reported *p*-values are below the significance level of α=0.05, indicating that the performance improvements achieved by the proposed method remain statistically significant under this stricter run-level evaluation across repeated trainings. On both datasets, M8 consistently outperforms the baseline methods in terms of paired run-level RMSE, supporting that the observed accuracy improvements are attributable to algorithmic advantages rather than random variation from a single training outcome.

Notably, in the test set comparison against the strongest baseline M7 (RPSO-DCFNN), although M7 shows competitive performance in some runs ($R^{-}=2$), the paired run-level analysis still yields a statistically significant difference (p=0.0051<0.01), suggesting that the proposed Neuro-Symbolic framework provides a more reliable calibration solution and can further reduce residual errors compared with the strongest baseline.

#### 3.5.7. Data-Efficiency

We further examined how calibration accuracy changes with the number of training samples. Specifically, we fixed the 100-sample test set and randomly downsampled the original 300-sample training set to $N_{\mathrm{train}}\in \left\{50,100,150,200,250,300\right\}$. For each $N_{\mathrm{train}}$, we repeated the subsampling 10 times with different random seeds and report the mean ± standard deviation of the test RMSE.

Table 11 shows that the error increases for small training sets, especially when $N_{\mathrm{train}}\leq 100$, which indicates insufficient workspace coverage to effectively constrain the learned residual field. This behavior is consistent with the principles of Physics-Informed Neural Networks (PINNs) discussed in recent studies [28,29]. These works demonstrate that embedding domain knowledge into the learning architecture provides essential regularization, which maintains physical consistency and enhances data efficiency in modeling complex nonlinear dynamics. In our framework, the symbolic D-H model acts as a rigorous physical anchor, allowing the adaptive wavelet networks to achieve performance saturation with as few as 250 to 300 samples. This suggests that our scale of data provides adequate coverage for the ABB IRB 120 workspace while minimizing the time required for industrial data collection.

#### 3.5.8. Cross-Platform Validation

To strengthen external validity beyond the ABB IRB120 platform, we additionally evaluated our framework on a cross-platform dataset, *HSR-RobotCali*, collected on an HSR JR680 industrial robot. The dataset contains 2000 samples evenly distributed across the workspace; each sample records the six joint angles and the measured cable length under the same acquisition protocol. This validation is conducted in an offline re-fitting setting without additional data collection. Specifically, we re-fit the model on *HSR-RobotCali* using an 80/20 train–test split and the same model architecture and hyperparameters as in the IRB120 study. Since *HSR-RobotCali* provides 1D distance measurements, we report the held-out test performance in terms of cable-length residual metrics. The *HSR-RobotCali* dataset is publicly available at https://github.com/Lizhibing1490183152/HSR-RobotCali (accessed on 29 January 2026).

As shown in Table 12, the proposed method achieves the best overall performance on the independent JR680 platform. In particular, M8 reduces the test RMSE from 0.85 mm (strongest baseline M7) to 0.55 mm, corresponding to a 35.3% relative improvement. The maximum residual is also reduced from 1.63 mm to 0.99 mm (39.3% reduction), indicating improved suppression of worst-case outliers. Moreover, the low across-seed variability of RMSE (±0.03 mm) suggests that the performance gain is stable under different random initializations. These results support the cross-platform applicability of the proposed framework under the 1D distance-measurement paradigm.

## 4. Conclusions

To address the stringent high-precision calibration requirements of industrial robots, this study proposes a PSO-Driven Neuro-Symbolic State-Space Framework. The approach advances the calibration paradigm by integrating three core components: an autonomous PSO meta-optimization module, an Adaptive Wavelet Network (AWNN) utilizing Mexican Hat kernels for non-geometric error compensation, and a decoupled global refinement strategy using the Levenberg–Marquardt optimizer. Experimental validation on an ABB IRB 120 robot yielded a test RMSE of 0.73 mm, representing a 40.16% improvement over the standard LM algorithm and a 12.05% reduction in error compared to the state-of-the-art RPSO-DCFNN baseline. Furthermore, ablation studies and rigorous run-level statistical tests (p<0.01) confirm that the AWNN outperforms standard MLPs, and the decoupled strategy effectively prevents geometric parameter coupling.

While the decoupling strategy exhibits high reliability under nominal conditions, highly intertwined geometric and elastic effects (e.g., dynamic payload variations or slow thermal drifts) present a theoretical boundary. Currently, the assumption of stationary symbolic states is partially relaxed by the process noise covariance Q in the EKF, which tracks slow, random-walk geometric drifts. Yet, if extreme temperature changes or payloads cause severe elastic deformations that violate rigid-body assumptions, the current purely configuration-dependent representation $\Phi (q_{k})$ may become insufficient.

Therefore, several directions remain for future investigation. First, to maintain the robustness of the decoupling strategy under highly variable conditions, future extensions will incorporate load and temperature sensor data directly into the AWNN input state vector. Second, to generalize to multi-sensor systems or multidimensional observations (e.g., 6D Cartesian pose estimation), the scalar observation $z_{k}$ would be expanded to a multidimensional vector $z_{k}\in R^{m}$, with the symbolic Jacobian and network output expanded accordingly to perform holistic multi-source data fusion. Additionally, we plan to validate the framework’s generalization to different mechanical structures (e.g., parallel manipulators), extend the offline learning phase into an online continual learning mechanism to adapt to mechanical wear, and pursue FPGA-based edge deployment for real-time dynamic compensation.

## Figures and Tables

**Table 1:** Nominal values of the robot kinematic parameters.

Joint *i*	$\alpha_{i}$ (deg)	$a_{i}$ (mm)	$d_{i}$ (mm)	$\theta_{i}$ (deg)
1	−90	0	290	0
2	0	270	0	−90
3	−90	70	0	0
4	90	0	302	0
5	−90	0	0	0
6	0	0	72	0

**Table 2:** Workflow of the PSO-Driven Neuro-Symbolic State-Space Framework.

Procedure	Complexity *
**Input:** Measurement set $D={\left\{\left(q_{k},z_{k}\right)\right\}}_{k=1}^{N}$; Initial guess $x_{0}$ (initialized from Table 1); Search space Ω for PSO.	—
**Stage 0: PSO Meta-Optimization**	
Initialize particle swarm S with random hyperparameters	Θ(P)
**while** not converged **do**	$\times I_{pso}$
Run Stage I (Train) for each particle using current params	Θ(N)
Evaluate Fitness: J=RMSE(Innovation)	Θ(1)
Update particle velocities and positions	Θ(P)
**end while**	—
**Output:** Optimal Hyperparameters $H^{*}=\left\{Q,R,\eta ,\theta_{init}\right\}$	—
**Stage I: Recursive Neuro-Symbolic Inference**	
Initialize $x_{0|0}\leftarrow x_{0}$, $P_{0|0}$, $\theta_{wav}\leftarrow H^{*}$	Θ(1)
**for** k=1 to *N* **do**	×N
*// 1. Symbolic State Prediction*	—
$x_{k|k-1}=x_{k-1|k-1}$	Θ(n)
$P_{k|k-1}=P_{k-1|k-1}+Q$	$\Theta (n^{2})$
*// 2. Neuro-Symbolic Fusion Observation*	—
Wavelet Residual: $\delta z_{k}^{wav}=\Phi \left(q_{k};\theta_{wav}\right)$	Θ(d)
Innovation: $\varepsilon_{k}=z_{k}-\left(h\left(x_{k|k-1},q_{k}\right)+\delta z_{k}^{wav}\right)$	Θ(1)
*// 3. Symbolic State Update (EKF)*	—
Jacobian $H_{k}=\partial h/\partial x$ (Symbolic only, see Equation (18))	Θ(n)
$S_{k}=H_{k}P_{k|k-1}H_{k}^{T}+R$	$\Theta (n^{2})$
$K_{k}=P_{k|k-1}H_{k}^{T}S_{k}^{-1}$	$\Theta (n^{2})$
$x_{k|k}=x_{k|k-1}+K_{k}\varepsilon_{k}$	Θ(n)
$P_{k|k}=\left(I_{24}-K_{k}H_{k}\right)P_{k|k-1}$	$\Theta (n^{2})$
*// 4. Adaptive Wavelet Update (SGD)*	—
Loss $L_{k}=\varepsilon_{k}^{2}$	—
Update $\left\{w,a,b\right\}\leftarrow \left\{w,a,b\right\}-\eta \cdot \nabla L_{k}$	Θ(d)
**end for**	—
**Output:** Symbolic Prior $x_{prior}$; Freeze $\theta_{wav}^{*}=\theta_{wav}^{\left(N\right)}$	—
Stage II: Decoupled Global Refinement (Frozen $\Phi^{*}$)	
Initialize $x\leftarrow x_{prior}$, $\lambda \leftarrow \lambda_{0}$; Define $\Phi^{*}\left(q\right)≜\Phi \left(q;\theta_{wav}^{*}\right)$	Θ(n)
**while** not converged **do**	$\times T_{\max}$
*// Step 1: Evaluation (Fixed Wavelet Field)*	—
r←0, J←0	—
**for** k=1 to *N* **do**	×N
Residual: $r_{k}=h\left(x,q_{k}\right)+\Phi^{*}\left(q_{k}\right)-z_{k}$	Θ(1)
Jacobian row: $J_{k}=\partial h/\partial x$	Θ(n)
**end for**	—
*// Step 2: Update (Standard LM)*	—
Solve $\left(J^{T}J+\lambda I\right)\Delta x=-\;J^{T}r$	$\Theta (n^{3})$
Update x and λ based on gain ratio (Equation (32))	Θ(n)
**end while**	—
**Final Output:** Optimal parameters $x_{opt}$, Wavelet Compensator $\Phi^{*}$	—
* *n*: symbolic state dim (24); *d*: wavelet network size; *P*: particle count.	

**Table 3:** Specifications of the draw-wire displacement sensor HY150-2000.

Item	Specification
Signal output type	Digital signal
Supply voltage	DC 5–24 V
Measuring range	2000 mm
Maximum speed	1000 m/s
Extension force	5 N
Linearity	0.05% FS
Resolution	0.004 mm
Operating temperature	−25∼+85 °C

**Table 4:** Hyperparameter settings for comparative calibration methods (M1–M7) and the proposed Neuro-Symbolic Framework.

Method	Hyperparameter	Value
M1 (EKF)	Initial state covariance $P_{0}$	$0.1\cdot I_{24}$
	Process noise covariance Q	$10^{-6}\cdot I_{24}$
	Measurement noise variance *R*	0.1
M2 (PF)	Number of particles $N_{p}$	1000
	Process noise standard deviation $\sigma_{w}$	$10^{-4}$
	Resampling threshold $N_{\mathrm{eff}}$	$0.5N_{p}$
	Maximum iterations	100
M3 (PSO)	Swarm size	50
	Inertia weight ω	0.7
	Cognitive coefficient $c_{1}$	1.5
	Social coefficient $c_{2}$	1.5
	Maximum iterations	200
M4 (RBFNN)	Number of hidden units	32
	RBF width σ	0.5
	Learning rate	$10^{-3}$
	Training epochs	500
M5 (LM)	Initial damping factor $\lambda_{0}$	$10^{-3}$
	Adjustment factor ν	10
	Convergence threshold	$10^{-8}$
	Maximum iterations	100
M6 (ANN-BFPA)	Population size *N*	40
	Switch probability *p*	0.8
	Levy flight parameter α	1.5
	Maximum iterations	500
M7 (RPSO-DCFNN)	RPSO Particle count *N*	2400
	RPSO Max iterations *M*	500
	RPSO Inertia weight *w*	0.9→0.4
	DCFNN Learning rate η	0.01
	DCFNN Max epochs $j_{\max}$	80
**M8 (Ours)**	*Stage 0: Meta-Optimization (PSO)*	
	Swarm size/Max iterations	30/50
	Search range for Q (log-scale)	[−8,−2]
	*Stage I: Adaptive Wavelet Network*	
	Wavelet kernel function	Mexican Hat
	Hidden neurons/Learning rate η	32/$10^{-3}$
	Optimized Process noise Q	$10^{-6}\cdot I_{24}$
	Optimized Measurement noise *R*	0.1
	*Stage II: Decoupled Refinement*	
	LM damping factor $\lambda_{0}$/ν	$10^{-3}$/10

**Table 5:** Performance comparison of various calibration algorithms (Mean ± Std over 10 random seeds).

	Training Set (300 Samples)			Test Set (100 Samples)		
Method	RMSE (mm)	STD (mm)	MAX (mm)	RMSE (mm)	STD (mm)	MAX (mm)
Before	4.26	4.34	5.31	5.81	5.78	6.84
M1 (EKF)	1.20±0.00	1.35±0.00	2.31±0.00	1.32±0.00	1.31±0.00	1.65±0.00
M2 (PF)	0.69±0.08	0.67±0.07	1.52±0.12	1.60±0.11	1.57±0.10	1.95±0.13
M3 (PSO)	0.74±0.06	0.65±0.05	1.31±0.11	1.04±0.07	1.00±0.05	1.31±0.13
M4 (RBFNN)	1.31±0.08	1.39±0.09	1.73±0.11	1.39±0.14	1.28±0.09	1.54±0.10
M5 (LM)	0.45±0.00	0.56±0.00	0.91±0.00	1.22±0.00	1.21±0.00	1.54±0.00
M6 (ANN-BFPA)	0.56±0.05	0.42±0.04	1.09±0.07	1.01±0.08	0.99±0.03	1.14±0.07
M7 (RPSO-DCFNN)	0.44±0.03	0.41±0.02	1.02±0.04	0.83±0.03	0.79±0.02	1.06±0.06
M8 (Ours)	0.26±0.01	0.24±0.02	0.79±0.02	0.73±0.01	0.68±0.01	0.99±0.02

**Table 8:** Extended ablation study regarding modules, kernels, and strategies.

Variant	Training Set			Test Set		
	RMSE (mm)	STD (mm)	MAX (mm)	RMSE (mm)	STD (mm)	MAX (mm)
V1: Symbolic Baseline	0.35	0.32	0.85	0.85	0.82	1.15
V2: w/o PSO (Rand)	0.30	0.38	0.95	0.77	0.85	1.25
V3: MLP Substitution	0.28	0.27	0.82	0.79	0.76	1.08
V4: Kernel (Morlet)	0.27	0.26	0.80	0.76	0.72	1.05
V5: Joint Optimization	0.28	0.29	0.88	0.92	0.88	1.18
V6: Proposed (Full)	0.26	0.24	0.79	0.73	0.68	0.99

**Table 9:** Wilcoxon signed-rank test results on the training set (10 repeated trainings; run-level RMSE).

Comparison	$R^{+}$	$R^{-}$	*p*-Value *
M8 vs. M1 (EKF)	55	0	0.0020
M8 vs. M2 (PF)	55	0	0.0020
M8 vs. M3 (PSO)	54	1	0.0039
M8 vs. M4 (RBFNN)	54	1	0.0039
M8 vs. M5 (LM)	53	2	0.0051
M8 vs. M6 (ANN-BFPA)	52	3	0.0098
M8 vs. M7 (RPSO-DCFNN)	52	3	0.0098
* The significance level is α=0.05 (two-sided Wilcoxon signed-rank test; exact *p*-values, N=10).			

**Table 10:** Wilcoxon signed-rank test results on the test set (10 repeated trainings; run-level RMSE).

Comparison	$R^{+}$	$R^{-}$	*p*-Value *
M8 vs. M1 (EKF)	55	0	0.0020
M8 vs. M2 (PF)	55	0	0.0020
M8 vs. M3 (PSO)	54	1	0.0039
M8 vs. M4 (RBFNN)	54	1	0.0039
M8 vs. M5 (LM)	53	2	0.0051
M8 vs. M6 (ANN-BFPA)	52	3	0.0098
M8 vs. M7 (RPSO-DCFNN)	53	2	0.0051
* The significance level is α=0.05 (two-sided Wilcoxon signed-rank test; exact *p*-values, N=10).			

**Table 11:** Effect of training sample size on calibration accuracy (fixed 100-sample test set). Mean ± std over 10 random subsamplings. The setting used in our method is 300 training samples. Bold indicates the best result.

Training Samples	50	100	150	200	250	300
**Test RMSE (mm)**	1.85±0.33	1.12±0.21	0.89±0.23	0.81±0.12	0.76±0.05	0.73±0.01

**Table 12:** Performance comparison on the *HSR-RobotCali* dataset (Mean ± Std over 10 random seeds). Bold indicates the best performance.

Method	RMSE (mm)	STD (mm)	MAX (mm)
M1 (EKF)	0.96±0.00	0.82±0.00	1.86±0.00
M2 (PF)	1.62±0.05	1.52±0.04	2.44±0.03
M3 (PSO)	1.14±0.05	1.00±0.03	2.05±0.02
M4 (RBFNN)	1.07±0.05	0.91±0.05	1.64±0.07
M5 (LM)	1.15±0.00	0.95±0.00	2.03±0.00
M6 (ANN-BFPA)	1.04±0.03	0.87±0.02	1.69±0.05
M7 (RPSO-DCFNN)	0.85±0.03	0.66±0.02	1.63±0.05
M8 (Ours)	0.55±0.03	0.42±0.03	0.99±0.06

## Data Availability

The cross-platform validation dataset *HSR-RobotCali* used in this study is publicly available at https://github.com/Lizhibing1490183152/HSR-RobotCali (accessed on 29 January 2026). The ABB IRB 120 experimental data supporting the reported results are available from the corresponding author upon reasonable request.
